# Evaluation of antibiotic prophylaxis use in a university dental clinic in the Lisbon area

**DOI:** 10.1080/07853890.2021.1897459

**Published:** 2021-09-28

**Authors:** Sara Manso, José João Mendes, Patrícia Cavaco Silva

**Affiliations:** ^a^Centro de Investigação Interdisciplinar Egas Moniz (CiiEM), Egas Moniz Cooperativa de Ensino Superior, Caparica, Portugal

## Abstract

**Introduction:**

Antibiotic prophylaxis (AP) in dentistry is recommended before dental procedures that involve manipulation of gingival tissue or periapical region, and perforation of oral mucosa in order to prevent a serious bacterial infection. The American Heart Association (AHA) Guideline recommends that the cardiac conditions with the highest risk of infective endocarditis (IE), for which AP is reasonable are: prosthetic cardiac valves, previous IE, congenital heart disease and cardiac transplantation with cardiac valvulopathy [1,2,3]. The aim of this study is to evaluate the use of AP in a University dental practice compared to AHA guidelines.

**Materials and methods:**

An observational and descriptive study was conducted between June and August 2016 at Clínica Dentária Egas Moniz (CDEM), a University Dental Clinic in the greater Lisbon Area. A total of 4000 patient records were analysed. 60 records were selected according to predefined inclusion criteria: patients submitted to endodontics and/or periodontology and/or surgery appointments, patients with IE/prosthetic cardiac valves/cardiac bypass/cardiac valvular disease/rheumatic fever or hip/knee joint prosthesis and patients who required AP before an invasive dental procedure. Clinical processes analysis were authorised by the patients through a declaration of informed consent. This study was authorised by the Clinical Director of CDEM and approved by Egas Moniz Ethics Committee.

**Results:**

Periodontology patients had AP indication in: congenital cardiac disease (4/16), cardiac valvular disease (5/13), rheumatic fever (3/7), and prosthetic cardiac valves (7/8). Surgery patients had AP indication in: IE (2/2), congenital cardiac disease (4/12), cardiac valvular disease (4/6), rheumatic fever (2/4) and prosthetic cardiac valves (6/8). Endodontic records were not enough to have significant results to compare.

**Discussion and conclusions:**

There is some disparity between the AHA guideline and the attitudes of dentists towards AP indication [4]. The use of antibiotics in dental medicine is characterised by empirical prescription based on epidemiological and clinical factors, using broad spectrum antibiotics and for short periods of time. The abuse of unjustified antibiotics and the lack of knowledge about the application of AP ultimately encourages bacterial resistance and the increase in untreatable infections. Antimicrobial resistance is a current serious global threat accordingly to WHO. It is no longer a prediction for the future, as it is happening in all regions of the world and has the potential to reach anyone [5]. The knowledge and communication over this topic must be stressed, even more in newly graduated dentists, to encourage evidence based homogeneous prescription patterns that promote good and safe clinical practices.
